# Modest genetic influence on bronchodilator response: a study in healthy twins

**DOI:** 10.3325/cmj.2015.56.152

**Published:** 2015-04

**Authors:** David Laszlo Tarnoki, Emanuela Medda, Adam Domonkos Tarnoki, Andras Bikov, Zsofia Lazar, Corrado Fagnani, Maria Antonietta Stazi, Kinga Karlinger, Zsolt Garami, Viktor Berczi, Ildiko Horvath

**Affiliations:** 1 Department of Radiology and Oncotherapy, Semmelweis University, Budapest, Hungary; 2 National Centre for Epidemiology, Surveillance and Health Promotion, Istituto Superiore di Sanità, Rome, Italy; 3 Department of Pulmonology, Semmelweis University, Budapest, Hungary; 4 The Methodist Hospital, DeBakey Heart & Vascular Center, Houston, TX, USA; * Each author contributed equally to this paper.

## Abstract

**Aim:**

To determine the reasons for large standard deviation of bronchodilator response (BDR) and establish whether there is a potential heritable component in healthy subjects.

**Methods:**

67 monozygotic and 42 dizygotic adult twin pairs were assessed for bronchodilator response (%change in FEV_1_ after inhaling 400 µg salbutamol). Univariate quantitative genetic modeling was performed.

**Results:**

Multiple regression modeling showed a significant association between BDR and sex and baseline FEV1 (*P* < 0.05), while no association was found with smoking habits, body mass index, or age. Within pair correlation in monozygotic twins was modest (0.332), but higher than in dizygotic twins (0.258). Age-, sex-, and baseline FEV1-adjusted genetic effect accounted for 14.9% (95% confidence interval, CI 0%-53.1%) of the variance of BDR, shared environmental effect for 18.4% (95% CI 0%-46.8%), and unshared environmental effect for 66.8% (95% CI 46.8%-88.7%).

**Conclusion:**

Our twin study showed that individual differences in BDR can be mostly explained by unshared environmental effects. In addition, it is the first study to show low, insignificant hereditary influences, independently from sex, age, and baseline FEV1.

The assessment of the reversibility of airway obstruction is a key element in the diagnosis of airway diseases and can also be a sign of the potential therapeutic effect of a specific inhaled drug (1,2). Bronchodilator response (BDR) is tested after the inhalation of a drug and is defined as the change in spirometric parameters including forced vital capacity (FVC), forced expiratory volume in 1 second (FEV_1_), and mid-expiratory flow 25%-75%. Although no consensus exists, the latest American Thoracic Society (ATS)/European Respiratory Society (ERS) guideline recommends that, during a single testing session, an increase in FEV_1_ or FVC>12% and ≥200 mL from baseline is considered a significant BDR (3).

Large population studies proposed a different threshold of BDR that has a role in clinical interpretation of airway response to a bronchodilator (4,5). However, underlying reasons for the large variation of BDR and a potential heritable component in healthy population are still unclear. It is known that certain genetic polymorphisms are associated with BDR in asthmatic (6,7) and non-asthmatic (8,9) individuals. This is supported by the fact that there are differences in BDR among different nations (10). These studies strongly support a hereditary influence on BDR, but they cannot fully describe the relationship of hereditary and environmental factors on the development of BDR. Assessing familial aggregation of BDR, Niu et al found a modest familial clustering (11).

Family design can determine inter-generation resemblance or difference, but, in contrast to the twin study design, it does not determine the influence of outside factors such as family, environment, and culture (12). Accordingly, family studies cannot reliably distinguish the influence of heritability from common environmental effects. Numerous twin studies examined airway hyperresponsiveness (AHR) (13-18), but none of them investigated BDR. Although BDR is usually associated with AHR in obstructive airway diseases (19), previous twin results on AHR cannot simply be extended to BDR. Therefore, the first aim of this study is to determine which factors affect BDR in a healthy twin population. As a second aim we investigated whether the large standard deviation of BDR in healthy asymptomatic subjects could be attributable to genetic or environmental factors.

## Participants and methods

### Participants

One hundred and nine adult twin pairs (67 monozygotic [MZ] and 42 dizygotic [DZ]) were recruited from the Hungarian Twin Registry (20) as part of the International Twin Study 2009. Zygosity was assessed using a standard validated questionnaire concerning the degree of physical similarity of twins during infancy. Zygosity determination through this method is >99% accurate (21). The study protocols were approved by the institutional review board for human studies of Semmelweis University, Budapest, Hungary (Regional and Institutional Committee of Science and Research Ethics, 29/2009) and written consent was obtained from each participant or their surrogate.

### Study design

Participants were recruited from general population via email, telephone, mail, or during twin festivals. Exclusion criteria included pregnancy, acute respiratory infection within three weeks of measurement, chronic obstructive airway diseases including asthma and chronic obstructive pulmonary disease (COPD), or foreseeable lack of compliance with test procedures. Participants were tested in two large hospitals in Budapest, Hungary by the same trained personnel (DLT, ADT). Prior to testing, they were asked to refrain from smoking for at least 3 hours, drinking alcohol or coffee for 10 hours, and eating for 1 hour.

### Baseline lung function measurement

Lung function was assessed by dynamic spirometry (Minispir, Waukesha, WI, USA). The spirometer was calibrated daily using a 1-L syringe. FEV_1_ measurements were performed in accordance with guidelines of the American Thoracic Society/European Respiratory Society Task Force (22).

### Bronchodilator response

Following the baseline lung function assessment, all participants inhaled 400 µg salbutamol in line with the ATS/ERS guidelines (22). BDR was assessed by bronchodilator response (change in FEV_1_ after inhaling 400 µg salbutamol). Salbutamol was inhaled from a metered dose inhaler without a spacer device and post- bronchodilator spirometry was recorded after 15 minutes. Bronchial reversibility was considered if the increase in FEV_1_ was at least 12% and 200 mL (23). No side effects were observed following the BDR tests.

### Statistical analysis

Descriptive statistics (mean ± standard deviation for continuous variables, percentage for categorical variables) were computed for MZ and DZ twins separately. MZ and DZ subsamples were compared by *t* test for paired samples. Multiple regression analysis identified potential variables associated with BDR, which were included as covariates in subsequent twin structural equation models. Regression analysis used robust estimation of standard errors to adjust for the clustering of data between twin and co-twin within pair.

Twin resemblance within MZ and DZ pairs was evaluated using Pearson correlation coefficients obtained with a saturated model in the Mx software (24); the saturated model was specified by constraining means and variances to be the same for twin and co-twin, and for MZ and DZ twins. A higher correlation in MZ than in DZ pairs suggests a contribution of genetic factors to the phenotype, similar correlation in MZ and DZ pairs suggests a contribution of shared environmental factors, while low correlation in MZ pairs compared to DZ twins suggests a contribution of unshared environmental factors. In particular, the genetic component is approximated by twice the difference between MZ and DZ correlation (2(MZ-DZ)) and the unshared environmental component is approximated by 1-MZ correlation (24,25).

Biometric univariate structural equation models were fitted to estimate the relative importance of hereditary and environmental effects on percent variation in FEV_1_ values (using the % change in FEV_1_ as a continuous variable) (24,26). These models incorporate latent variables for genetic and environmental influence and observed variables for measured FEV_1_ change in twins. The effect of latent variables on observed variables is inferred from the observed variances and covariances by exploiting the fact that MZ twins share 100% of their genes, while DZ twins share 50% of the genetic background. Twin models assume that relevant environmental exposures are shared by the twins to the same extent regardless of zygosity (“equal environments assumption”) (24).

Using these models, it is possible to decompose the phenotypic variance of BDR into additive genetic (A), shared environmental (C), and unshared environmental (E) components (ACE model). Shared environment includes those factors that are not related to individual lifestyle, such as familiar socialization, air pollution, shared womb, while unique environment includes smoking or nutrition. All twin analyses were adjusted by age, sex, and baseline FEV1. The analyses were carried out with Stata (version 11.0, StataCorp, College Station, TX, USA) and Mx softwares (Department of Psychiatry Virginia Institute for Psychiatric and Behavioral Genetics, Richmond, VA, USA) (27). The level of statistical significance was set at *P* < 0.05.

## Results

61.5% of twins were MZ and 38.5% were DZ (72% women) (Table 1). No significant difference between the groups was observed in age, body mass index, and smoking prevalence. DZ twins had higher FEV_1_ change compared to MZ twins, although the difference was not significant (3.4 ± 6.5 vs 2.0 ± 5.5%, *P* = 0.278). 11 participants had a positive BDR test. Although BDR (FEV_1_ change) showed no correlation with baseline FVC (L) (*P* > 0.05), a significant but weak inverse correlation was noted with FEV_1_/FVC (r = -0.20, *P* < 0.001). Participants with FEV_1_/FVC<0.70 (n = 13) showed a greater BDR response than participants with FEV_1_/FVC > 0.70 (8.6 vs 2.0, *P* < 0.01).

**Table 1:** Characteristics of twins according to zygosity. Data reported as mean ± standard deviation or n (%)*

	Total	Monozygotic	Dizygotic	*P*
Twin subjects, n (%)	218	134 (61.5)	84 (38.5)	-
Male, n (%)	61 (28.0)	32 (23.9)	29 (34.5)	0.550
Age, years	44.7 ± 15.6	44.3 ± 15.9	45.4 ± 15.2	0.788
Body mass index, kg/m^2^	25.8 ± 4.9	25.5 ± 4.9	26.3 ± 4.9	0.361
Never smokers, n (%)	149 (69.0)	95 (72.0)	54 (64.3)	0.354
Past smokers, n (%)	31 (14.3)	16 (12.1)	15 (17.9)	
Current smokers, n (%)	36 (16.7)	21 (15.9)	15 (17.9)	
Positive BDR test, n (%)^†^	11 (5.1)	4 (3.0)	7 (8.3)	0.430
FVC, l	3.6 ± 0.9	3.4 ± 0.9	3.8 ± 1.0	
FVC, % predicted	3.7 ± 0.9	3.6 ± 0.8	3.8 ± 1.0	0.124
FEV_1_, l	3.0 ± 0.8	2.9 ± 0.7	3.1 ± 0.8	0.156
FEV_1_, % predicted	3.1 ± 0.8	3.0 ± 0.7	3.2 ± 0.8	0.067
FEV_1_/FVC	0.84 ± 0.08	0.82 ± 0.11	0.83 ± 0.09	0.767
Mean FEV_1%_ change	2.5 ± 6.0	2.0 ± 5.5	3.4 ± 6.5	0.278

*BDR – bronchodilator response; FVC – forced vital capacity; FEV_1_ – forced exhaled volume in one second.

†defined as 12% and 200 mL increase in FEV_1_ following 400 µg salbutamol.

In multiple robust regression analysis, smoking status, body mass index, and age were not significantly associated with BDR. Biometric models were adjusted for age as well as for variables that were associated with FEV_1_ change in the regression model (ie, sex and baseline FEV_1_) (Table 2).

**Table 2:** Multiple regression analysis of FEV_1_ (forced exhaled volume in one second) % change and considered covariates. Full model*

FEV_1%_ change	Coefficient	Standard error	*P*	95% confidence interval
Sex (males vs females)	-0.567	0.239	0.018	-1.036, -0.099
Age	-0.151	0.095	0.110	-0.336, 0.034
Baseline FEV_1_	-0.317	0.126	0.012	-0.563, -0.069
Smoking habits^†^				
past smokers	-0.179	0.226	0.428	-0.622, 0.264
current smokers	-0.187	0.204	0.360	-0.589, 0.214
Body mass index	-0.06	0.089	0.477	-0.239, 0.111

*All continuous variables were standardized to have zero mean and a standard deviation of 1 prior to estimating the regression equation.

†Reference category: never smokers.

Intraclass correlation of percent change in FEV_1_ was modest but higher in MZ twins (*r*MZ = 0.332, 95% CI 0.083, 0.532) than in DZ twins (*r*DZ = 0.258, 95% CI -0.041, 0.499), suggesting a weak genetic effect in the expression of BDR. ACE analysis indicated that genetic and shared environmental effects were modest and that they together explained about one-third of total variance in FEV_1_ change (A:15%, C: 18%), while unshared environmental influence explained the largest part of variance (E: 67%) (Table 3). In the ACE model, sex and baseline FEV_1_ were significantly associated with FEV_1_ change, while age, BMI, and smoking history were not significantly associated (data not shown).

**Table 3:** Within pair correlations and genetic and environmental variance components of FEV_1_ (forced exhaled volume in one second) % change as estimated under the ACE model (A – heritability; C – shared environment; E – unshared environment). Numbers in parentheses indicate 95% confidence intervals*†

Measure	Twin correlation		Proportions (%) of variance components		
	rMZ	rDZ	A	C	E
FEV_1%_ change	0.332 (0.083, 0.532)	0.258 (-0.041, 0.499)	14.9 (0, 53.1)	18.4 (0, 46.8)	66.8 (46.8, 88.7)

*FEV_1_ – forced exhaled volume in one second; rMZ – correlation in monozygotic twins; rDZ – correlation in dizygotic twins.

†Intraclass correlations and variance components were adjusted for age and sex and baseline FEV_1_.

## Discussion

Although the assessment of bronchodilator response has become routine in clinical practice, background of the large variation of BDR in asymptomatic persons has remained unclear. BDR response is a common, but not a specific measure of bronchial asthma, which has considerable prevalence among non-asthmatic persons (28). We found that BDR was weakly influenced by genetic factors and shared environment, and strongly influenced by unshared environmental factors. This result might clarify the large standard deviation of BDR responses in asymptomatic persons without airways disease. The finding that unshared environmental component mainly explains the variation also suggests the importance of prevention (eg, smoking prevention in persons with high-risk for asthma/COPD or lifestyle modification). Accordingly, our results highlight that lifestyle factors have an important role in the determination of BDR test results in individuals without lung diseases, so these factors should be eliminated before BDR assessment and be taken into account in the interpretation of the results.

Genetic influences have been investigated in diseases related to pathologic BDR. For example, heritability of asthma has been widely demonstrated (29-34). Although the current twin study is the first study investigating BDR, numerous workgroups examined airway hyperresponsiveness, another common characteristic of asthma (13-18). Hopp et al suggested the role of hereditary influences on airway hyperresponsiveness to methacholine by simply estimating the MZ and DZ correlations for twin pairs (6-31 years) (15). They also reported that increased non-specific bronchial reactivity was persistent and associated with allergy (14). Zamel et al demonstrated in ten MZ and ten DZ healthy, nonsmoking twins the major role of environmental factors in determining the variability of acute AHR to bronchoactive drugs (18). An Australian twin study in 381 twin pairs aged 8 to 18 years suggested that hereditary effects may be largely shared between asthma, atopy, and AHR (13). Lund et al reported no heritability for AHR in younger adult twins (age 18-31 years) (16) and that shared environmental factors explained 30% of the AHR variance (16). These factors may include diet, exposure to high levels of air pollution, parental smoking and other conditions in the parental home, such as house dust mites, mold, etc. Compared to their results, we found a weak hereditary component for BDR (15%) and a similar (67%) unshared environmental component. Unshared environmental factors may include differences in diseases and occupational exposures. This is supported by a previous study reporting a relationship between bronchial hyperresponsiveness and infections after the neonatal period in school-aged twins (35). Apart from twin studies, there is evidence from familial aggregation studies, segregation analyses, linkage studies, and genome-wide linkage analyses of hereditary influence on BDR, suggesting that interactions between genes and environmental components may also be involved (13,15,36-40).

Smoking status is an important environmental factor to be examined. Although smoking increases the risk of airway responsiveness (36), Lund et al in their heritability analysis did not analyze the effect of smoking (16). In the present study, the twin sample comprised 32.9% active or ex-smokers. Although the association of smoking with AHR is well known (41), we did not observe a significant role of smoking status on BDR; however, this may be explained by the relatively low rate of active smokers (17.3%). Accordingly, the effect of smoking on the ACE models was not assessed. The lack of relationship between smoking status and BDR is not surprising, as a study conducted in a large cohort of patients with obstructive airway disease showed that BDR was related to smoking history rather than to current smoking status (19). However, we could not completely eliminate the possible effects of smoking on ACE modeling by stratifying the quantitative genetic analyses according to pair-wise smoking status and analyzing the non-smokers only. This limitation should be considered in future larger twin studies investigating the effect of smoking on the heritability of BDR. Structural equation modeling has decreased power to assess models (for genetic vs shared environmental vs unique environmental components) when the genetic effect is low (less than ~ 20%) (42). This also prevented the exploration of sex differences in the heritability and environmental effects on BDR.

The main strength of the present study is that all BDR tests were performed by the same trained researchers and the same validated device at all sites. Furthermore, the results can be generalized to the non-twin adult population, assuming that the enrolled twins did not differ from non-twin individuals with regard to the considered traits.

In conclusion, the present study revealed a low heritability of BDR independently of sex, age, and baseline FEV_1_ in a healthy twin population. Unshared environmental effects explained most of the BDR variance, underscoring the importance of environmental factors (eg, lifestyle, allergens) in determining individual differences in BDR in healthy adult individuals. This finding might explain the frequently experienced large standard deviation of BDR assessments in asymptomatic persons without airways disease, emphasizing the importance of lifestyle factors that influence BDR test results.
